# Progress in public health risk communication in China: lessons learned from SARS to H7N9

**DOI:** 10.1186/s12889-019-6778-1

**Published:** 2019-05-10

**Authors:** Melinda Frost, Richun Li, Ronald Moolenaar, Qun’an Mao, Ruiqian Xie

**Affiliations:** 1 Division of Global Health Protection, Center for Global Health, Centers for Disease Control and Prevention, Atlanta, GA USA; 2 US Centers for Disease Control and Prevention, Beijing, China; 3 National Health Commission, Beijing, China; 4 Chinese Center for Health Education, Beijing, China

**Keywords:** Global health security, Risk communications, China, International health regulations, Global disease detection, SARS, H7N9

## Abstract

**Background:**

Following the SARS outbreak, the World Health Organization revised the International Health Regulations to include risk communication as one of the core capacity areas. In 2006, the U.S. Centers for Disease Control and Prevention’s Global Disease Detection [GDD] program began collaborating with China to enhance China’s risk communication capacity to address gaps in the SARS communication response. This article describes tangible improvements in China’s public health emergency risk communication capacity between the SARS and H7N9 outbreaks; documents U.S. CDC GDD cooperative technical assistance during 2006–2017; and shares lessons learnt to benefit other countries and contribute to enhance global health security.

**Method:**

A questionnaire based on the WHO Joint External Evaluation tool [Risk Communication section] was developed. A key communications official from the China National Health Commission [NHC] completed the questionnaire retrospectively to reflect China’s capacity to manage communication response before, during and after the outbreaks of SARS in 2003, influenza H1N1 in 2009, and influenza H7N9 in 2013. A literature search was also conducted in English and Chinese to further substantiate the results of the questionnaire completed by NHC.

**Results:**

China demonstrated significantly improved risk communication capacities of pre-event, during event and post event responses to H7N9 when compared to the SARS response. China NHC improved its response through preparedness, availability of dedicated staff and resources for risk communication, internal clearance mechanisms, standard operating procedures with national response parties external to NHC, rumor management, communication with international agencies and consistent messaging with healthcare and private sectors. Correspondingly, the perceived level of trust that the public had in the NHC following outbreaks rose between the SARS and H7N9 response.

**Conclusion:**

Risk communication capacities in China have increased during the ten years between the SARS outbreak of 2003 and the H7N9 outbreak of 2013. Long-term risk communication capacity building efforts in bilateral collaborations are uncommon. The U.S. CDC GDD project was one of the first such collaborations worldwide. The lessons learned from this project may benefit lower and middle-income countries as they build their national emergency risk communication capacity.

**Electronic supplementary material:**

The online version of this article (10.1186/s12889-019-6778-1) contains supplementary material, which is available to authorized users.

## Background

In 2003, the world was struck by the epidemic of a new viral disease, transmitted by direct contact and respiratory droplets. The Severe Acute Respiratory Syndrome (SARS) resulted in 8096 cases and 774 deaths reported to the World Health Organization (WHO). In addition to the enormous human costs of SARS, it extracted an estimated $ 40–54 billion USD from the global economy [1]. Of over 25 affected countries, China experienced the greatest burden, with 5327 cases and 349 deaths [2].

The WHO referenced a list seven lessons learned from SARS. Among the lessons was that global health security relies on the capacity of all countries to rapidly detect and contain public health threats at their source. Another lesson learned regarded the importance and challenge of risk communication [3] during a public health emergency. Following the SARS outbreak, WHO revised the International Health Regulations [IHR]. Among the revised IHR, risk communication was included as one of the core capacity areas [4]. WHO defines risk communication as

*"... the real-time exchange of information, advice and opinions between experts or officials and people who face a threat [hazard] to their survival, health or economic or social well-being. Its ultimate purpose is that everyone at risk is able to take informed decisions to mitigate the effects of the threat [hazard] such as a disease outbreak and take protective and preventive action.*

*Risk communication uses many communications techniques ranging from media and social media communications to mass communications and stakeholder and community engagement. It requires the understanding of stakeholder perceptions, concerns and beliefs, as well as their knowledge and practices. Effective risk communication must also identify early on and subsequently manage rumours, misinformation and other communications challenges"* [5]*.*

In 2006, a bilateral ministerial level Memorandum of Understanding (MOU) was jointly signed by the Minister of Health of China and the U.S. Secretary of Health and Human Services (HHS). Under the framework of this MOU, the Emerging and Re-emerging Infectious Disease Collaborative (EID) program was officially launched, which marked the initiation of U.S. Centers for Disease Control and Prevention’s (U.S. CDC) Global Disease Detection (GDD) program’s collaboration with China. This is a comprehensive collaboration between the U.S. and China, including multiple projects focusing on various subject areas. A health communication capacity building project was initiated with the goal to enhance China’s public health risk communication capacities in an effort to address some of the challenges identified during the SARS outbreak. U.S. CDC has provided cooperative technical assistance for risk communication capacity to China for over a decade. A bilateral communication capacity building program of this scale is a rare occurrence globally, and lessons learned from this experience may be of value to countries building their emergency risk communication capacity as well as to technical assistance partners providing this capacity expertise.

From 2006 until present, the GDD risk communication program has worked with Chinese public health agencies such as the National Health Commission (NHC - formerly Ministry of Health), the Chinese Center for Disease Control and Prevention (China CDC) and the Chinese Center for Health Education (CCHE). Program areas focused on risk communication guideline development and implementation, training for public health professionals including spokespeople, health emergency responders, health educators and health officials in charge of emergency response in all 31 provincial level regions in mainland China.

While China struggled in response to SARS in 2003, it improved its capacity; as a result, China’s response to another potentially pandemic virus, 2013 Influenza A (H7N9), has been described as a model. This paper aims to: describe tangible improvements in China’s public health emergency risk communication capacity between SARS and H7N9; provide process outcome data and anecdotes; document U.S. CDC GDD cooperative technical assistance during this time period and to 2017; share lessons learned to benefit other countries and provinces; and suggest next steps to improve risk communication globally, and thereby contribute to enhanced global health security.

## Methods

We used an established assessment method developed by WHO to explore the changes in China’s risk communication capacity. In 2016, the WHO IHR Review Committee adopted a new assessment method and tool known as the Joint External Evaluation (JEE). The JEE tool and process are “intended to assess country capacity to prevent, detect, and rapidly respond to public health threats independently of whether they are naturally occurring, deliberate, or accidental” [6]. The JEE tool provides a more detailed series of questions to measure a country’s capacity to communicate effectively with its public according to five domains: 1) Risk Communication Systems [plans, mechanisms], 2) Internal and Partner Communication and Coordination, 3) Public Communication, 4) Communication Engagement with Affected Communities and 5) Dynamic Listening and Rumour Management.

Questions were selected from each of the five JEE risk communication domains as well as globally accepted risk communication principles of trust, timeliness, transparency, listening to audiences, and planning [7]. The questions were adapted and asked of a NHC key communication official to retrospectively determine China’s capacity to manage communication response before (pre-event), during (event) and after (post-event) the outbreaks of SARS in 2003, influenza H1N1 in 2009, and influenza H7N9 in 2013. The SARS and H7N9 outbreaks occurred approximately 10 years apart and serve as appropriate communication response comparisons as well as bookends to much of the risk communication capacity building efforts between U.S. CDC and China.

These binary and Likert scale questions were asked of Director Mao Qun’an, one of the few senior communications officials and spokespersons in China’s NHC and its predecessor the Ministry of Health (MOH) that was directly involved in the SARS and H7N9 outbreaks and all public health emergencies in between these two events. As our key informant, Director Mao quantified capacity and provided documented and anecdotal support for his results. Following Director Mao’s response, a literature search was conducted in English and Chinese to further substantiate the results of his feedback regarding China’s communication response capacity. Capacity building efforts between U.S. CDC GDD and China were mapped on a timeline and reviewed for potential impact and lessons learned.

## Results

The original study intended to look at response capacity change from SARS in 2003 to H1N1 in 2009 and H1N1 to H7N9 in 2013. While the greatest change in capacity occurred following SARS and in time for the H1N1 response, we chose to focus on the entire body of capacity change between SARS and H7N9 noting that no key lessons learned have been eliminated by merging these results.

### Risk communication capacity growth from SARS to H7N9

#### Pre-event capacity

“Public communication during an outbreak represents an enormous challenge for any public health authority and therefore demands sound planning, in advance, to adhere to the principles described above. Planning is an important principle, but more importantly, it must translate into action” [7]. Of the three response phases [pre-event, event and post-event] the greatest increase of risk communication capacity occurred for the pre-event capacities between the SARS and H7N9 outbreaks. Considering the JEE risk communication domain of systems and plans, prior to SARS, there was no risk communication or emergency response plan. Therefore, response agencies had no agreed-upon mechanisms stating which individuals would be responsible for what actions. In the case of risk communication coordination, there was no guidance on who would communicate what information when and through what channels to best inform the public in a timely and transparent fashion.

During the SARS response, there were no staff dedicated to emergency risk communication and therefore no prior training for staff. There were also no agreements for internal clearance of messages and no shared plans or agreements between health authorities and other response partners. Lacking these coordinated approaches led to severe delays in information release or lack of release at all and in some cases conflicting information from several authorities. However, by the time of the H7N9 outbreak, the NHC/MOH denoted that communications preparedness efforts such as plans, budgets and a dedicated and trained communication staff including a spokesperson were in place. Mechanisms for NHC/MOH internal clearances and sharing of standard operating procedures with national response parties external to the NHC/MOH were established. The perceived level of trust among the public for the NHC/MOH prior to an emergency event was higher before H7N9 as compared to SARS responses (Additional file 1).

The NHC addressed internal and external coordination issues by establishing cross-cutting response mechanisms. The Department coordination system of NHC synchronizes 31 different relevant departments that all play a part in emerging infectious disease response. The system focuses on information sharing, prevention activities, training and drills [8]. These activities and an ongoing coordination effort better prepared China prior to the next public health emergency.

#### Event capacity

Among the documented failures of China’s handling of the early stages of the outbreak was the restriction of information to the public [9]. “China’s delayed detection of the outbreak and – in particular – its poor level of communication during the response to the emergency probably led to many avoidable cases of SARS and damaged China’s economy and reputation” [10]. Approximately half of the risk communication response capacities during these public health emergencies were somewhat in place at the time of SARS, but again improved greatly between the SARS and H7N9 outbreaks (Additional file 2). At the time of SARS, NHC/MOH lacked mechanisms to coordinate communication among national and international responders and stakeholders. This improved between the outbreak responses potentially due to the implementation of IHR in the interim. Other proactive capacities such as audience analyses, message testing, and frequent media updates proved to be lacking capacities during the SARS outbreak but were used as part of the communications response for the H7N9 outbreak and H1N1 before that.

Rumours, misinformation, or misperceptions can plague communication response efforts. In an age where social media proliferates information rapidly, media monitoring and rumour management are necessities in any communication response effort [11]. Rumour management and shifts in communication response based on audience feedback were better utilized during outbreaks following SARS. Echoing a better communications response, NHC felt its communication to be more trustworthy, transparent and timely for the H7N9 outbreak response.

Most of the event capacity indicators were positive for the H7N9 communication response. There was more of an emphasis on sharing information with international agencies and coordinating with healthcare and private sectors. There was also an increased focus on audience analysis and engagement with affected communities. The risk communication system within NHC/MOH seemed to become more sophisticated as assurance of coordinated and consistent messaging between response agencies improved between outbreak responses. The perceived level of transparency among members of the public as well as the perceived level of speed in which information was released to the public rose (Additional file 2).

In order to better coordinate response among health agencies, the NHC established the Office of Responses to Public Health Emergencies. This serves as the Headquarters for Response to Public Health Emergencies and is responsible for integrating resources [8]. China invested in its communication system and the efforts were rewarded with a recognized coordinated effort both in terms of its early and rapid communication to the public and coordination between response sectors such as public and animal health agencies [10]. Another joint study done by the EID health communication capacity building project team (U.S. CDC and CCHE) showed through a series of focus groups that the public’s view of China’s H7N9 response was positive. They appreciated the timely and transparent communication and both trusted and followed the public health recommendations [12].

#### Post-event capacity

Lessons learned from SARS and later communication responses were integrated into the NHC/MOH communication plans, shared with the public and partners and used to improve outreach to target audiences. Following SARS, it appears as though the NHC/MOH began to monitor the effectiveness of its messages and to respond to rumours. Overall, lessons learned were used to improve future communication response efforts. The NHC/MOH increased its ability to manage rumours by monitoring and evaluating response to determine that either behaviors were changed or rumours stopped due to NHC/MOH action. Correspondingly, the perceived level of trust that the public had in the NHC/MOH following outbreaks rose between the SARS and H7N9 response (Additional file 3).

A study of early communication and response in southern mainland China showed that the quick and well-coordinated response led to relatively high levels of trust in the Chinese government advice [13].

While the change in risk communication capacity indicators signified an improved response on the part of China, some may question the adoptability of western-based principles into the Chinese or any other non-western culture. A study that the EID conducted compared audience reactions to messages written about a hypothetical disease outbreak. One set of messages were written prior to risk communication training and the other after training. Results showed that reading the post training messages audiences felt reduced anxiety typically associated with uncertainty, increased sense of control, increased trust with public agencies, and a sense that the communication was transparent [14] (Additional file 4).

### Risk communication technical assistance provided from 2006 to 2017

Following SARS, there were a number of risk communication technical assistance activities primarily coordinated by U.S. CDC, the WHO, and its Western Pacific Regional Office (WPRO). The majority of cooperative activities occurred through the EID program.

Early collaborative activities of health communication capacity building project occurred between U.S. CDC and its assigned Chinese counterpart, China CDC. Throughout the course of the technical assistance program, collaborative partners such as the NHC/MOH and CCHE were added. The program shifted course slightly from year to year in an effort to establish a base of risk communication adoption at provincial and sub-provincial levels while trying to influence policy and emergency risk communication response systems at the national level. The early years of the program were dedicated to partnering with China CDC. There, the collaboration helped to build a nascent risk communication capacity where previously there had just been a two-person media team. The efforts began to build foundational knowledge of risk communication nationally through workshops taught with provincial and sub-provincial health bureaus and local CDCs.

By 2008, there was enough underlying capacity to begin formalizing into an emergency risk communication system. This highlighted the fact the NHC/MOH was the true mouthpiece of the health sector during a public health emergency, not China CDC. While it was valuable to teach the epidemiologic technical staff of China CDC and the Field Epidemiology Training Program (FETP) to communicate transparently, rapidly and with empathy, these principles had to be recognized and adopted at the top of the health sector. Coordination and partnership continued as the program continued to support capacity building efforts of CCHE at national and sub-national levels and included FETP and hospital responders in joint capacity building sessions.

Also, in 2008, EID supported a MOH/China CDC delegation’s visit to U.S., focusing on health emergency risk communication related information and experience exchange. The delegation composed of an MOH spokesperson, representatives from health emergency response groups in both MOH and China CDC, senior epidemiologist from China CDC and key staff from the CCHE. They first visited the U.S. CDC headquarters to see its emergency communication system in operation, then visited Washington, D.C. to see how the system interoperated with the Health and Human Services system, and lastly visited a state-based system to observe the operations at state and local levels. This event, later followed by numerous fellowship programs at U.S. CDC, resulted in recognition from the Minister of Health to institute risk communication as a critical function in China’s public emergency preparedness and response efforts.

The Chinese delegation chronicled their trip in an article written for the Chinese public. The article stated, “In recent years, promoted by MOH, risk communication concepts and theories have been gradually introduced into and accepted by the Chinese health system. Relevant technical guidelines and training materials have been developed. More public health professionals and officials have realized the importance of risk communication, which is a critical component of the entire public health emergency response system. However, risk communication has also frequently been mistaken with health communication and other concepts. There is still a big gap in comprehensive and correct understanding about risk communication systems and mechanisms and detailed practices. Therefore, it’s an important and urgent need for us to establish a public health emergency risk communication mechanism fitting China’s situation and based upon the U.S. public health emergency risk communication mechanism and experiences. We should make risk communication a standardized institutionalized technical function, which will help control and decrease hazards caused by public health emergencies” [15].

#### Public health emergency risk communication guideline and handbook

All of these activities likely contributed to China’s increased risk communication strategy but the government itself claims that a few efforts particularly made a difference. The development and distribution of the Public Health Emergency Risk Communication Guideline and later a step-by-step handbook significantly increased awareness and improved risk communication skills for Chinese public health emergency response workers. The development of the Public Health Emergency Risk Communication Guideline is regarded as the initiation of risk communication theory and practice in Chinese health administration departments [16]. This also created the expectation that risk communication should be included in emergency response preparedness plans. Health emergency response experts were seen as potential spokespeople and included in training workshops. This highlighted the important role risk communication plays in emergency response; it also provided inspiration and a model for the development of a Chinese language national public health emergency training textbook [17]. The MOH distributed 10,000 copies of the Public Health Emergency Risk Communication Guideline and 15,000 copies of the Handbook to health agencies [including provincial health bureaus-health emergency response offices, provincial CDC-health emergency response offices, public health inspection institutes, health education institutes and some hospitals] across China.

#### Provincial emergency risk communication training workshops

Provincial training workshops provided assistance and guidance for risk communication capacity building in each of the 31 mainland provinces in China. These sessions remarkably increased awareness and skills for internal and cross-sector communications, collaboration with media, and communicating with the general public. The workshops promoted the inclusion of risk communication into routine public health emergency response trainings as a critical component. Risk communication is also now included in the provincial health emergency response preparedness plan. Workshop participants regularly commented that the training content was very relevant to their actual work duties.

The health communication capacity building project team conducted a study to determine risk communication knowledge, practice and/or barriers to risk communication practice among public health practitioners in China. Results showed that while some barriers such as lack of autonomy at provincial levels still occurred, practitioners had an awareness of risk communication practice due to training. “Findings of this assessment confirm that risk communication training efforts by the Chinese NHC/CCHE and U.S. CDC have been successful in developing awareness of risk communication principles among public health practitioners and their ability to implement those principles in practice” [18].

#### Communication - a more visible force in National Health Commission and China CDC

Over the course of the past ten years, communication has moved from a function that largely served as a mechanism to release boilerplate statements from the MOH to a non-investigative media with little nuanced information for audience segments, to meaningful recommendations mindful of personal barriers and empathy for affected audiences. Now, there are visible functions within China CDC and the NHC/MOH that are tasked with a spectrum of communication functions to better reach the public with timely and transparent information that citizens need to protect their health. In 2013, the Department of Communication was established when the previous MOH was reorganized into the current NHC. Working with media and releasing information on behalf of NHC are included as part of its key responsibilities The main functions include “drafting goals, plans, policies and standards for … public health education and health promotion, … , news and information release” [19].

#### Feedback from Chinese counterparts

Chinese counterparts have provided feedback that the risk communication capacity building project introduced risk communication concepts and theories into China. It increased awareness and the skills of public health emergency response workers and their roles in the communication process. The project also allowed for the inclusion and recognition of risk communication as a critical component of health emergency response work in China. It introduced risk assessment and risk communication into emergency preparedness plans as well as routine health emergency response work. However, plans don’t work if the leaders don’t articulate and practice the principles of risk communication. Chinese counterparts also recognized that the education provided at both national and provincial levels provided a common language and set of expectations to health bureau and education staff that resulted in improved nationwide practice. At the same time, provincial spokespersons across China gained a voice steeped in the practice of risk communication [15] (Additional file 5).

## Discussion

Risk communication capacity in China has increased impressively. The most dramatic demonstration of this increased capacity is depicted in the difference between China’s response to SARS in 2003 and its response to H7N9 in 2013. A number of actions likely contributed to the work China has done to improve its emergency risk communication capacity. These include more defined IHR capacity indicators that included risk communication, the technical assistance and support of international partnerships such as U.S. CDC and WHO, and most of all the willingness and aspirations of thousands of public health workers and leaders in China to communicate more rapidly and transparently to the public. According to Mao Qun’an, “Chinese health authorities thoroughly realized the importance of risk communication and have continually worked to improve response plans, evaluation methods and personnel training. Risk communication has played a more and more important role in public health emergency response and has contributed to positive outcomes in emergency response as well.”

Long-term risk communication capacity building efforts in bilateral collaborations are uncommon. The EID program health communication capacity building project was one of the first such collaborations worldwide. Through the experience of working with China in the time period following SARS, a number of lessons were learned. Lower and middle income countries and their capacity building programs can benefit from the knowledge gained and thus build on these experiences to further the science of national emergency risk communication capacity building.

### Lessons learned in risk communication capacity building

#### Seek the voice of public health emergencies

From 2006 to 2009, the U.S. CDC risk communication activities primarily partnered with China CDC. At the time, the organization did not have the ability to speak on behalf of China’s public health system to the public during emergencies. Realizing the need to not only communicate with the public but also ensure that immediate responders and field epidemiologists could effectively work with communities, the U.S. CDC realigned its partnership with both the China CDC and NHC/MOH. This allowed China CDC to build its communication capacity at the local levels to better engage with communities and share information in a more effective way [rapidly, conscious of barriers to health protection practices, using terminology better understood by the public, and choosing communication channels regularly used by the affected population]. This information was shared up to the NHC/MOH level where national messages were then broadcast to the public.

#### Once you’ve found the voice, find the eyes and ears of your audiences

The EID program health communication capacity building project aimed to support and test a number of communication channels. The program supported public health agencies’ use of social media to reach its audiences, research on effectiveness of SMS messaging during emergencies, the national “12320” hotline to ensure consistent and timely answers to the public's questions, user friendly emergency communication websites for China CDC, and health education programming by co-producing “12320” web-chats featuring U.S. and Chinese public health experts.

The projects increased in complexity and reach and were typically coupled with a methodology of testing a new media or method that the Chinese counterpart agency hadn’t yet experienced with promotion of timely health topics of interest to the public and/or aligned with a “health” day. As an example, the “12320” web-chats included topics such as rabies, tuberculosis on World TB day, seasonal influenza vaccination at the beginning of flu season, and Ebola at the height of the West Africa outbreaks. This channel had the potential audience size of 4 million. The cooperation also supported China CDC’s and NHC/MOH’s effective use of social media and rumour monitoring and management to address misinformation and misperception rapidly during emergencies such as H1N1. Finally, to test the efficacy of SMS use for messaging the population during H1N1, the cooperation tested messages during the outbreak finding that SMS can improve knowledge gain and influence attitudes but didn’t affect behavioral changes except for some self-reported short-term behaviors such as vaccine uptake [20].

#### Don’t limit communications capacity building to communicators only

Through the experience of working with China’s public health system in addition to numerous other more recent risk communication capacity building experiences, we learned to work beyond our direct communications counterparts. Integration of risk communication practices requires acceptance at all levels of an organization's hierarchy, use during all phases of an emergency and actions on the part of responders not typically considered to be ‘communicators’. As was the case in China, acceptance of risk communication principles required behavior change on the part of policy makers and leaders. Because emergencies require different communication strategies at different response phases, leaders must realize the importance of communication to ensure that communicators are part of the earliest phases of a response before it becomes problematic to mitigate issues caused by delayed or ineffective communication.

Finally, as was the case with the Chinese Field Epidemiology Training Program (CFETP), field epidemiologists often don’t consider themselves communicators. As of March 2017, the CFETP program had graduated 288 epidemiologists, 95% of whom were working in China’s public health system [21]. China, like other countries supporting an FETP, found that a number of its FETP graduates are becoming influential public health leaders. During our risk communication training workshops with CFETP officers, some would comment that they couldn’t necessarily use risk communication practice because, in their positions, they were not allowed to speak to the media about sensitive information such as outbreak case numbers before being authorized by health administrative departments. Temporarily setting aside the fact that risk communication doesn’t only use media communication, our immediate response was, “you may not be able to talk to the media now, but you are the future of China’s public health system. There’s a likelihood that you will be a leader in this system and therefore someday you will talk to the media. Now, let’s make sure you do that well.”

Risk communication includes not only outreach through media but also two way communication to affected populations [7]. Community engagement is increasingly being recognized as a critical function in any outbreak setting [22]. FETP officers are often among the first public health responders during outbreaks and emergencies. Information about the public health emergency and information to the affected population starts locally at the site of the incident. Therefore, FETP becomes a critical link in the communication chain by providing guidance to the affected population and detailed analysis of the situation to regional and national decision makers and eventually communicators that distribute information more broadly to the media. FETP officers are in a position to communicate to affected individuals in a rapid, transparent and empathetic manner which should better enable greater trust between the public and public health authorities. With an understanding of proper messaging to the public, field epidemiologists can craft their communication to leaders and communicators in ways that help public comprehension and lessen confusion - a worthwhile contribution to any emergency response.

#### Train communicators as part of a response force

Working with the CFETP over a number of years and knowing that U.S. CDC’s Epidemic Intelligence Service contains officers with a variety of backgrounds and skills, EID piloted a program to include four communicators as part of the CFETP. The communication officers were tasked to respond to outbreaks and develop provincial risk communication plans alongside their counterparts in the provincial health bureaus, CDCs and hospital systems and also response to emergencies. The realization that communicators are needed as part of a field response is also increasingly recognized at U.S. CDC and at WHO. In 2013, WHO initiated a global version similar to this program called the Emergency Communications Network [23].

#### Scaling up and across in China

China is the world’s most populous country with approximately 1.374 billion people [24] in a landmass nearly the size of the U.S. To change risk communication policy, it’s natural to work with national level ministries, but for practice to be well absorbed into the health response system throughout the nation it had to be adopted at provincial and sub-provincial levels.

Additionally, risk communication has to be coordinated among all response partners. Therefore, adoption of practices by just one agency wouldn’t have much, if any, effect. As mentioned, whenever possible, training workshops and exercises included health response partners from a variety of agencies that should coordinate during emergencies. Sometimes this happened automatically, particularly in the latter years of the cooperation, but other times it required some guidance. For the work through U.S. CDC GDD we would often support workshops for a certain organization [China CDC or MOH]. In many of the cooperative agreement documents, we would stipulate that one organization had to reserve a certain number of seats for a partnering organization. We would also regularly meet with our counterparts at the WHO office in Beijing to ensure that we were targeting technical assistance areas in a complimentary fashion as opposed to duplicating efforts or not addressing gaps.

#### A picture is worth a thousand words, experience is worth a million

The impact that the 2008 senior leadership delegation visit to the U.S. had on risk communication policy in China was unexpected. Having impassioned and influential partners witness for themselves how an effective public health communication system could interoperate on a political [HHS], technical [U.S. CDC] and sub-national [U.S. state and local] scale provided the vision they needed to implement risk communication practice in China. Additionally, U.S. CDC’s Emergency Risk Communications Branch hosted a number of Chinese counterparts as visiting fellows. These fellows are still actively involved in and in some cases leading risk communication functions in China CDC, NHC/MOH and CCHE.

## Conclusion

A timeline of risk communication technical assistance between U.S. CDC and the China CDC and the MOH/NHC depicts capacity building efforts growing and diversifying during the time period between SARS and H7N9. Capacity building efforts changed as needs changed and, in many cases, decreased. A search of Chinese literature was conducted to illustrate what capacity building efforts changed policy and improved preparedness and response capabilities. In addition, key informants in the MOH provided additional insights on systematic advancements in the Chinese public health risk communication system during and following capacity building efforts. Capacity increased along the same path as technical assistance.

Lessons learned from this bilateral technical assistance program can be adapted by other risk communication capacity programs. Ensuring that capacity is built with not only the “voice” of the public health system such as policy makers and leaders but also with responders such as FETP officers can best ensure institutional behavior change. Likewise, programs should train communicators on how to respond to emergencies at the field level where they’re needed. Encourage partnerships and cooperation. The more agencies and organizations that adopt risk communication practice and collaborate and coordinate during the preparedness phase, the more seamless the next emergency response will be. Showing dedicated and influential counterparts a working risk communication system can help clarify and inspire policy action.

Strengthening risk communication capacity is an important component of worldwide efforts to enhance global health security. IHR implementation, which is aimed at ensuring that all countries have the capacity to rapidly detect and contain public health threats at their source, requires that all countries have adequate and effective risk communication capacity. Lessons learned from the risk communication capacity building efforts in China were used to develop capacity assessment tools currently being used by IHR and may be helpful for other countries striving to implement IHR, and thereby can contribute to enhanced global health security.

The U.S. CDC GDD efforts should not be credited for the success of China’s improved ability to communicate both with its population and the global community during public health emergencies; that credit goes to the China CDC, the China Ministry of Health/National Health Commission, and the hardworking men and women serving in public health roles across the country.

## Additional files

Additional file 1: China Risk Comms Table 1 Pre event results 2017.docx (DOCX 14 kb)

Additional file 2: China Risk Comms Table 2 Event results 2017.docx (DOCX 15 kb)

Additional file 3: China Risk Comms Table 3 Post event results 2017.docx (DOCX 14 kb)

Additional file 4: China Risk Communication capacity then and now comparison Table 08312017.docx (DOCX 16 kb)

Additional file 5: China Risk Comms Cap Building timeline 20170831.docx (DOCX 29 kb)

## Abbreviations

CCHE: Chinese Center for Health Education
CFETP: China field epidemiology training program
China CDC: China center for disease control
EID: Emerging and re-emerging Infectious disease program
FETP: Field Epidemiology Training Program
GDD: Global Disease Detection
HHS: Health and Human Services
IHR: International Health Regulations
JEE: Joint External Evaluation
MOH: Ministry of Health (of China) Earlier name of the Chinese MOH
MOU: Memorandum of Understanding
NCH: National Health Commission (of China). (Current name of the former Chinese MOH)
SARS: Severe Acute Respiratory Syndrome
SMS: Short message service
U.S. CDC: U.S. Centers for Disease Control and Prevention
USD: US dollars
WHO: World Health Organization
WPRO: Western Pacific Regional Office
