# Supplementary material for: Progress in public health risk communication in China: lessons learned from SARS to H7N9
Source: BMC Public Health. 2019 May 10;19(Suppl 3):475. doi: 10.1186/s12889-019-6778-1 (PMC6696672; doi:10.1186/s12889-019-6778-1)
Supplement: Supplementary file 1 — China Risk Comms Table 1 Pre event results 2017.docx (DOCX 14 kb) [file 12889_2019_6778_MOESM1_ESM.docx]

# Table 1: Risk communication capacity pre-event questionnaire results (SARS and H7N9) using IHR/JEE questions

| **Pre-Event Capacity Question** | | **SARS** | **H7N9** |
| --- | --- | --- | --- |
|  | **Was there a national response plan prior to the __________ outbreak?** | **N** | **Y** |
|  | **If so, was risk communication included in the national response plan prior to the __________ outbreak?** |  | **Y** |
|  | **Was there a permanent staff dedicated to risk communication prior to the __________ outbreak?** | **N** | **Y** |
|  | **Was training provided to the risk communication personnel for response to local hazards prior to the __________ outbreak?** | **N** | **Y** |
|  | **Was there a dedicated budget line for communication personnel, materials and activities prior to the __________ outbreak?** | **N** | **Y** |
|  | **Was there an agreement internal to your agency for clearance of messaging to the public prior to the __________ outbreak?** | **N** | **Y** |
|  | **Were there shared communication plans, agreements and/or standard operating procedures between other response agencies such as public safety, law enforcement, hospitals, emergency response, Red Cross/Crescent and/or government agencies such as Ministries of Defence, Agriculture, Food/Drug, etc. prior to the __________ outbreak?** | **N** | **Y** |
|  | **Did your organization have a designated and trained spokesperson prior to the __________ outbreak?** | **N** | **Y** |
|  | **On a scale of 1 – 10 (1= no trust and 10= complete trust), what degree of trust do you believe the public had with your agency prior the __________ outbreak?** | **6** | **8** |
