# Supplementary material for: Progress in public health risk communication in China: lessons learned from SARS to H7N9
Source: BMC Public Health. 2019 May 10;19(Suppl 3):475. doi: 10.1186/s12889-019-6778-1 (PMC6696672; doi:10.1186/s12889-019-6778-1)
Supplement: Supplementary file 2 — China Risk Comms Table 2 Event results 2017.docx (DOCX 15 kb) [file 12889_2019_6778_MOESM2_ESM.docx]

# Table 2: Risk communication capacity event questionnaire results (SARS and H7N9) using IHR/JEE questions

| **Event Capacity Question** | | **SARS** | **H7N9** |
| --- | --- | --- | --- |
|  | **Was there a surge staff activated for risk communication during the __________ outbreak** | **Y** | **Y** |
|  | **Was there a mechanism to formally coordinate and communicate internally within your agency during the __________ outbreak**? | **Y** | **Y** |
|  | **Was there a mechanism to formally coordinate and communicate among national stakeholders and response agencies during the __________ outbreak?** | **N** | **Y** |
|  | **Was there a mechanism to formally coordinate and communicate among international stakeholders and response agencies during the __________ outbreak?** | **N** | **Y** |
|  | **Were there incidents where stakeholder/partner agencies released information that was inconsistent or contradicted your agency’s information during the __________ outbreak?** | **Y** | **N** |
|  | **Were there incidents where valuable time was taken because of a lack of agreement regarding which agency would be responsible for coordinating communication during the __________ outbreak?** | **Y** | **N** |
|  | **Was there a formal mechanism to coordinate communication with the health care sector (hospitals, both public and private) during the __________ outbreak?** | **Y** | **Y** |
|  | **Was there a formal mechanism to coordinate communication with the private sector during the __________ outbreak?** | **N** | **Y** |
|  | **Did your organization conduct target audience analysis to better understand audience language, trusted information resources and preferred communication channels during the __________ outbreak?** | **N** | **Y** |
|  | **Did your organization have a communication strategy that proactively reached out to a variety of media platforms such as newspapers, radio, TV, social media, web in order to target communication messages to specific audiences during the __________ outbreak?** | **N** | **Y** |
|  | **Did your organization test the key messages and materials before they were disseminated during the __________ outbreak?** | **N** | **Y** |
|  | **During the __________ outbreak, did your organization provide regular media briefings and updates through mass media and social media?** | **N** | **Y** |
|  | **Did your organization monitor for rumours and misinformation and when found address the issues rapidly during the __________ outbreak?** | **N** | **Y** |
|  | **Did your organization have a social mobilization, health promotion or community engagement department or working group that reached out to the affected or at risk populations during the __________ outbreak?** | **Y** | **Y** |
|  | **Did your organization have a social mobilization, health promotion or community engagement function working at intermediate (district/provincial) levels during the __________ outbreak?** | **Y** | **Y** |
|  | **Was there an ongoing and functioning feedback loop between at-risk or affected populations and response agencies during the __________ outbreak?** | **N** | **Y** |
|  | **Did your organization regularly and rapidly change messaging to address audience feedback, misinformation and questions during the __________ outbreak?** | **N** | **Y** |
|  | **Did your organization consider communication feedback including rumours and misinformation from the public in its decision making process to improve communication response during the __________ outbreak?** | **N** | **Y** |
|  | **Did your organization regularly share information on rumours and misinformation with other sectors of the emergency response, and with other communication partners during the __________ outbreak?** | **N** | **Y** |
|  | **On a scale of 1 – 10 (1= no transparency and 10= complete transparency), what degree of transparency was used to communicate to during the __________ outbreak?** | **5** | **9** |
|  | **On a scale of 1 – 10 (1= slow/none and 10= rapid), how fast was information released and available to the public during the __________ outbreak?** | **5** | **10** |
