# Supplementary material for: Progress in public health risk communication in China: lessons learned from SARS to H7N9
Source: BMC Public Health. 2019 May 10;19(Suppl 3):475. doi: 10.1186/s12889-019-6778-1 (PMC6696672; doi:10.1186/s12889-019-6778-1)
Supplement: Supplementary file 3 — China Risk Comms Table 3 Post event results 2017.docx (DOCX 14 kb) [file 12889_2019_6778_MOESM3_ESM.docx]

# Table 3: Risk communication capacity post-event questionnaire results (SARS and H7N9) using IHR/JEE questions

| **Post-Event Capacity Question** | | **SARS** | **H7N9** |
| --- | --- | --- | --- |
|  | **Were alterations made to response plans based on lessons learnt from the __________ outbreak?** | **Y** | **Y** |
|  | **Looking back on the event, were there significant improvements that could be made in the staffing, platforms, financial resources or other factors to improve communication with the public, partners and affected or at risk communities during the __________ outbreak?** | **Y** | **Y** |
|  | **Did your organization share experience and new strategies with partner organizations to continually improve communication response following the __________ outbreak?** | **Y** | **Y** |
|  | **Did your organization contribute to an evidence base of what communication methods are best to influence positive behaviours among target audiences following the __________ outbreak?** | **Y** | **Y** |
|  | **Did your organization conduct media research to determine message reach to target audiences following the __________ outbreak?** | **Y** | **Y** |
|  | **Did your organization monitor the effectiveness of methods or messages used to disprove a rumour or correct misinformation during the __________ outbreak?** | **N** | **Y** |
|  | **Did your organization regularly evaluate its communication response and ability to address rumours and misinformation to determine that actions changed behaviour and/or stopped the rumour from spreading during the __________ outbreak?** | **N** | **Y** |
|  | **On a scale of 1 – 10 (1= no trust and 10= complete trust), what degree of trust do you believe the public had with your agency following the __________ outbreak?** | **7** | **9** |
