# Supplementary material for: Progress in public health risk communication in China: lessons learned from SARS to H7N9
Source: BMC Public Health. 2019 May 10;19(Suppl 3):475. doi: 10.1186/s12889-019-6778-1 (PMC6696672; doi:10.1186/s12889-019-6778-1)
Supplement: Supplementary file 4 — China Risk Communication capacity then and now comparison Table 08312017.docx (DOCX 16 kb) [file 12889_2019_6778_MOESM4_ESM.docx]

| **Risk communication capacity then and now**  *A comparison of risk communication domain topics* | |
| --- | --- |
| **Risk Communication Capacity 2003** | **Risk Communication Capacity 2017** |
| **Risk Communication Systems (plans, mechanisms)** | |
| **2003**   - No national emergency response plan - No risk communication plan | **2017**   - Risk communication is integrated into a National Emergency Response Plan - A separate Emergency Risk Communication plan exists and is tested/used by the NHC and China CDC - Plans continue to improve based on lessons learned from past communication responses |
| **Internal and Partner Communication and Coordination** | |
| **2003**   - No internal coordination of information within Ministry of Health and sub-national entities - No sharing of information between government response partners - No information clearance chain | **2017**   - NHC department coordination system synchronizes 31 relevant response departments - Office of Responses to Public Health Emergencies established to better coordinate response internal to NHC - NHC, China CDC and CCHE all coordinate public communication horizontally at the national level and vertically with sub-national entities - Regular sharing of information with international agencies and coordinating with healthcare and private sectors |
| **Public Communication** | |
| **2003**   - Restricted or delayed information to the public - No or little use of media channels that the public uses | **2017**   - Rapid information sharing with the public - Frequent media updates and information on where to seek addition information - Use of multiple media channels with consistent messaging |

| **Communication Engagement with Affected Communities** | |
| --- | --- |
| **2003**   - No identification of specific/segmented audiences - No analysis of audience information needs or preferred communication channels - No testing of messages for comprehension or potential barriers to action - No specific engagement with communities | **2017**   - Audience analyses regularly conducted prior to emergencies - Message testing conducted prior to and during emergencies - Mechanisms exist to engage with communities |
| **Dynamic Listening and Rumour Management** | |
| **2003**   - Media monitoring of reputational issues vs public health threats - No action taken to mitigate the negative public health effects of rumours and misinformation | **2017**   - Regular media monitoring for rumours, misinformation and misperceptions - Mechanisms in place to change communication strategy according to information learned through media monitoring and rumour management |
