# Supplementary material for: Progress in public health risk communication in China: lessons learned from SARS to H7N9
Source: BMC Public Health. 2019 May 10;19(Suppl 3):475. doi: 10.1186/s12889-019-6778-1 (PMC6696672; doi:10.1186/s12889-019-6778-1)
Supplement: Supplementary file 5 — China Risk Comms Cap Building timeline 20170831.docx (DOCX 29 kb) [file 12889_2019_6778_MOESM5_ESM.docx]

*Figure 1 Timeline of Risk Communication capacity building collaborative activities between U.S. CDC and China CDC, NFHPC/MOH and CCHE in China 2006-2012*
